# Cancer incidence during the COVID‐19 pandemic by region of residence in Manitoba, Canada: A cancer registry‐based interrupted time series study

**DOI:** 10.1002/cam4.6698

**Published:** 2023-11-16

**Authors:** Kathleen M. Decker, Allison Feely, Oliver Bucher, Piotr Czaykowski, Pamela Hebbard, Julian O. Kim, Harminder Singh, Maclean Thiessen, Marshall Pitz, Grace Musto, Katie Galloway, Pascal Lambert

**Affiliations:** ^1^ Paul Albrechtsen Research Institute CancerCare Manitoba Winnipeg Manitoba Canada; ^2^ Department of Community Health Sciences, Max Rady College of Medicine, Rady Faculty of Health Sciences University of Manitoba Winnipeg Manitoba Canada; ^3^ Department of Epidemiology and Cancer Registry CancerCare Manitoba Winnipeg Manitoba Canada; ^4^ Department of Internal Medicine, Max Rady College of Medicine, Rady Faculty of Health Sciences University of Manitoba Winnipeg Manitoba Canada; ^5^ Department of Medical Oncology and Hematology CancerCare Manitoba Winnipeg Manitoba Canada; ^6^ Department of Surgery, Section of General Surgery, Max Rady College of Medicine, Rady Faculty of Health Sciences University of Manitoba Winnipeg Manitoba Canada; ^7^ Department of Radiology, Section of Radiation Oncology, Max Rady College of Medicine, Rady Faculty of Health Sciences University of Manitoba Winnipeg Manitoba Canada; ^8^ Department of Radiation Oncology University of Manitoba Winnipeg Manitoba Canada

**Keywords:** cancer, COVID‐19 pandemic, incidence, region

## Abstract

**Introduction:**

Health care in Manitoba, Canada is divided into five regions, each with unique geographies, demographics, health care access, and health status. COVID‐19‐related restrictions and subsequent responses also differed by region. To understand the impact of the pandemic on cancer incidence in the context of these differences, we examined age‐standardized cancer incidence rates by region over time before and after the COVID‐19 pandemic.

**Methods:**

We used a population‐based quasi‐experimental study design, population‐based data, and an interrupted time series analysis to examine the rate of new cancer diagnoses before (January 2015 until December 2019) and after the start of COVID‐19 and the interventions implemented to mitigate its impact (April 2020 until December 2021) by region.

**Results:**

Overall cancer incidence differed by region and remained lower than expected in Winnipeg (4.6% deficit, 447 cases), Prairie Mountain (6.9% deficit, 125 cases), and Southern (13.0% deficit, 238 cases). Southern was the only region that had a significantly higher deficit in cases compared to Manitoba (ratio 0.92, 95% CI 0.86, 0.99). Breast and colorectal cancer incidence decreased at the start of the pandemic in all regions except Northern. Lung cancer incidence decreased in the Interlake‐Eastern region and increased in the Northern region. Prostate cancer incidence increased in Interlake‐Eastern.

**Conclusions:**

The impact of the COVID‐19 pandemic on cancer incidence differed by region. The deficit in the number of cases was largest in the southern region and was highest for breast and prostate cancers. Cancer incidence did not significantly decrease in the most northern, remote region.

## Graphical abstract

The impact of the COVID‐19 pandemic on cancer incidence differed by region in Manitoba, Canada. The deficit in the number of cases was largest in the southern region and was highest for breast and prostate cancers. Cancer incidence did not significantly decrease in the most northern, remote region.

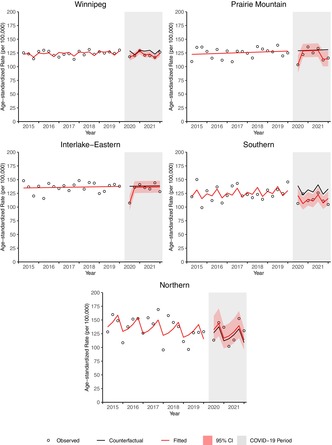

## 1 INTRODUCTION

The first COVID‐19 case in Manitoba was identified on March 12, 2020. In response, the government of Manitoba quickly implemented province‐wide restrictions to mitigate the impact of the virus, including limiting the number of individuals who could gather, suspending primary, secondary, and post‐secondary school classes, closing the Canada–US border to non‐essential travel, and requiring a mandatory 14‐day self‐isolation for people entering Manitoba from other provinces.^1^ Because of concerns that the impact of COVID‐19 would be greater among communities in northern Manitoba due to factors ranging from a higher prevalence of chronic health conditions, food insecurity, and overcrowded housing,^1^, ^2^ on April 17, 2020, the government also issued public health orders that restricted travel to northern Manitoba. Additional restrictions were implemented for the southern region of the province on October 5, 2021, because of lower vaccination rates and higher case counts.^3^ These measures varied over time as COVID‐19 waves fluctuated.^4^ The cancer care system also implemented changes such as the greater use of virtual visits, the temporary suspension or reduction of cancer screening and diagnostic services, triaging patients for treatment based on acuity, and the redeployment of health care staff to process COVID‐19 tests and care for acute cases of COVID‐19.^5^ We previously examined cancer incidence for all of Manitoba and found a significant decrease early in the pandemic and sustained decreases throughout the pandemic for individuals 75 years of age and older with breast or lung cancer, as well as for urinary, brain, and CNS cancers.^6^

However, we hypothesize that the impact of the pandemic on cancer incidence might differ by regions within a province. COVID‐19 incidence was higher in some areas than others and societal responses to restrictions and vaccination varied markedly.^7^ For example, travel to northern Manitoba was limited to residents only, whereas other regions did not have this restriction. In southern Manitoba, there were lower rates of COVID‐19 vaccinations and higher infection rates, which may have disrupted health care more compared to other regions. To understand the impact of the pandemic on cancer incidence in the context of these differences and provide information about possible disparities based on where individuals live, we conducted a population‐based quasi‐experimental study that examined age‐standardized cancer incidence rates by region over time before and after the COVID‐19 pandemic. We also estimated the difference between the predicted (i.e., counterfactual estimates in the absence of COVID‐19) and fitted (i.e., smoothed predicted estimates of observed data) number of cancer cases for each region.

## 2 MATERIALS AND METHODS

### 2.1 Setting

**FIGURE 1:**
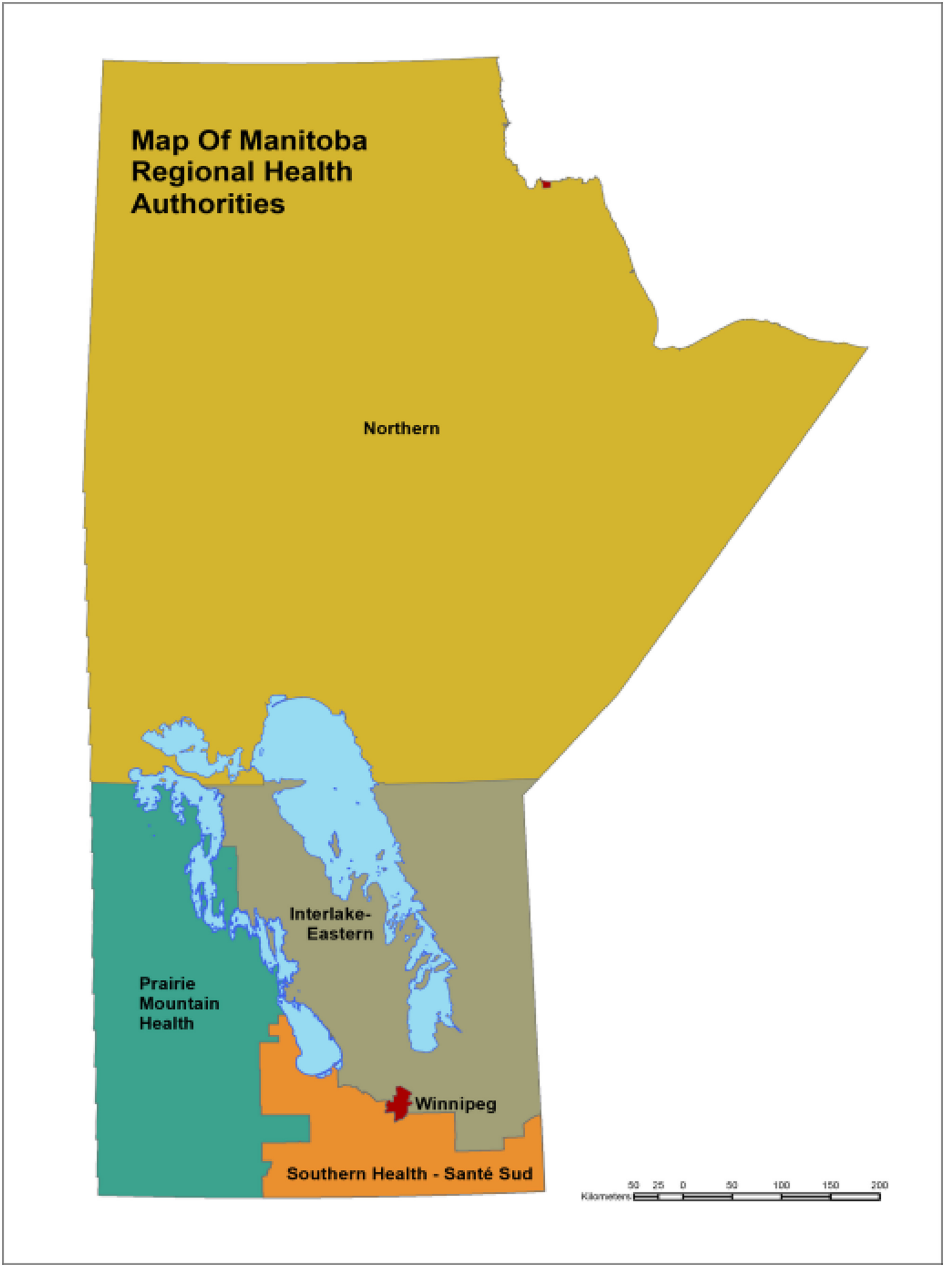
Map of Manitoba's regional health authorities.

Health care delivery in Manitoba (1.39 million as of 2020) is provided by five regional health authorities: the Winnipeg Regional Health Authority (https://wrha.mb.ca/, 648 km^2^, population 791,284, 57.1% of the population including the capital city of Winnipeg and the town of Churchill), the Interlake‐Eastern Regional Health Authority (https://www.ierha.ca/, 61,000 km^2^, population 133,834, 9.6% of the population, primarily rural), Prairie Mountain Health (https://prairiemountainhealth.ca/, 67,000 km^2^, population 172,641, 12.4% of the population, primarily rural), Southern Health‐Santé Sud (https://www.southernhealth.ca/, 27,025 km^2^, population 211,896, 15.3% of the population, primarily rural), and the Northern Health Region (https://northernhealthregion.com/, 396,000 km^2^, population 77,283, 5.6% of the population, the most northern and remote region) (Figure 1).^8^ Each regional health authority differs in terms of demographics, health care access and use, and population health status. Northern Health has the youngest population (37.8% 19 years of age or younger) while Prairie Mountain Health and the Interlake‐Eastern Regional Health Authority have the oldest populations (19.3% and 19.4% 65 years of age or older, respectively).^8^ The province's tertiary health care facilities are located in Winnipeg. Community hospitals, local clinics, and nursing stations provide health care throughout rural and northern Manitoba. Northern has the lowest social deprivation score of all health regions, which suggests strong relationships with families, workplaces, and communities but the poorest health status compared to the Manitoba average.^9^ Southern has the best overall health status.^9^

COVID‐19 case counts and societal responses to restrictions and vaccination also varied by regional health authority.^7^ Testing during the second and third waves was highest in Northern (1008 per 100,000 person‐years) and lowest in Southern and Prairie Mountain (442 and 450 per 100,000 person‐years, respectively).^10^ Hence, COVID‐19 incidence rates were highest in Northern (140.6 per 100,000 person‐years) and lower in Prairie Mountain (24.3 per 100,000 person‐years), Interlake‐Eastern (35.6 per 100,000 person‐years), and Southern (41.0 per 100,000 person‐years). As of April 2022, two‐dose vaccination rates were 82.9% for Manitoba and were lowest in Southern (66%) and highest in Winnipeg (88%).^9^ During the second wave before vaccination, the number of deaths per 100,000 was highest in Winnipeg.^11^ After vaccination was available, Southern accounted for 33% of the COVID‐19 related deaths in the province during the third wave despite comprising 15% of the provincial population.^11^

### 2.2 Design and population

We used a population‐based quasi‐experimental study design to examine the rate of new cancer diagnoses over time by quarter (January–March, April–June, July–September, and October–December) for before (January 2015 until December 2019) and after the start of COVID‐19 and the interventions implemented to mitigate its impact (April 2020 until December 2021) in Manitoba, Canada by region. The study was approved by the University of Manitoba's Health Research Ethics Board (HS23979; H2020:264) and CancerCare Manitoba's (CCMB) Research and Resource Impact Committee (2020‐14).

### 2.3 Data sources

The primary source of data was the Manitoba Cancer Registry (MCR). The MCR is a population‐based registry that is legally mandated to collect and maintain accurate, comprehensive information about cancer diagnoses in Manitoba and has consistently shown to be of very high quality.^12^ The MCR uses disease site groupings according to the International Classification of Diseases for Oncology, Third Edition (ICD‐O‐3) versions.

### 2.4 Outcomes

Outcomes included the age‐standardized cancer incidence rate per 100,000, the estimated cumulative difference in the number of new cancer cases, and the estimated percent cumulative difference in the number of new cancer cases by region of residence. Incidence was defined as the number of new cases of cancer diagnosed in the population during a time period. The estimated cumulative difference in new cancer cases was defined as the difference between the quarterly cumulative counterfactual count and the quarterly cumulative fitted count (i.e., the observed number of cases smoothed). The estimated percent cumulative difference in the number of new cancer cases was defined as the cumulative difference in the fitted count divided by the cumulative difference in the counterfactual count. Rates were examined for all cancers as well as specifically for breast, colorectal, prostate, and lung cancers (the four most common types of cancer diagnosed in Manitoba). eAppendix A in the supplement lists the ICD‐O‐3 codes. The region was based on Manitoba's five regional health authorities. The Winnipeg Regional Health Authority includes the town of Churchill located in northern Manitoba because residents of Churchill often travel to Winnipeg to receive health care. However, to reflect where an individual lives and not where they receive care, we have included Churchill as part of the Northern Health Region. Rates were age‐standardized using the direct method to the 2011 Canadian population. Population data were based on estimated Manitoba Health coverage provided by Manitoba Health.

### 2.5 Statistical analysis

We used an interrupted time series (ITS) analysis^13^ that takes into consideration the baseline trend for the incidence rate of cancers in the population. If the baseline trend is not considered, the difference between the number of cases observed and the number expected in the absence of COVID‐19 will be either overestimated (if there was a downward trend in cases prior to the pandemic) or underestimated (if there was an upward trend in cases prior to the pandemic). Therefore, we compared post‐COVID‐19 (April 2020 onward) cancer incidence rates to counterfactual rates as if the pandemic had not occurred based on pre‐COVID‐19 trends (baseline rates) and quantified quarterly new cancer diagnoses for all cancers as well as breast, colorectal, prostate, and lung cancer by area of residence.

Generalized linear models (GLMs) based on the distribution of the data were used for the analyses. First, the predicted mean and variance of each model and the observed mean and variance in the data were plotted.^14^ Scaled quantile residual plots were also used to evaluate the overall uniformity of residuals and dispersion.^15^ Each model included a binary intervention term that was equal to 0 during the pre‐COVID‐19 period and 1 during the COVID‐19 period and a time term defined as the number of quarters since the start of the study period. Binary variables for quarters 2, 3, and 4 were added to the models if they improved model fit to account for seasonality. Fitted values (i.e., predictions of observed values that were smoothed) were generated. Counterfactual values were generated by predicting values with the binary indicator of the pandemic as 0 instead of 1. Autocorrelation of residuals were evaluated.

After the appropriate GLM model was selected, models were fine‐tuned by comparing the adjusted McFadden *R* ^2^ between subsequent models.^16^ Plots were then produced using the observed, counterfactual, and fitted values. If the plotted counterfactual did not follow the baseline trend, the model was modified (i.e., the number of degrees of freedom for splines was reduced) until the counterfactual was consistent with the baseline trend. COVID‐19‐by‐time interactions were also considered if the plotted fitted values in the COVID‐19 period did not fit the observed data well (e.g., observed values were below fitted values during the early COVID‐19 period but higher during the later COVID‐19 period). Significant time interactions were found for some cancer sites but still demonstrated poor fit (i.e., the fitted observed values did not fit the observed values well). Therefore, we created multiple COVID‐19 dummy variables representing different periods during the pandemic to provide a slope for each period, which enabled more accurate predictions. The following R packages were used: haven, splines, Hmisc, lattice, MASS, ggplot2, car, DHARMa, multcomp, glmmTMB, lmtest, and forest plot.^17^, ^18^, ^19^, ^20^, ^21^, ^22^, ^23^, ^24^, ^25^ The first quarter of 2020 was excluded from the analyses because COVID‐19 restrictions as well as changes to visit and treatment protocols were implemented incrementally throughout March 2020.^5^, ^26^, ^27^

Ratios between counterfactual and fitted estimates and 95% confidence intervals (CIs) (derived from contrast statements) were calculated. The estimated cumulative difference in the number of cases during the pandemic was calculated as of December 31, 2021, and plotted using a forest plot. The 95% CIs for the cumulative difference estimates were calculated with parametric bootstrapping and 1000 replications. Data analyses were performed in SAS version 9.4 (SAS Institute Inc., Cary, NC, USA) and R version 4.0.5 (R Foundation for Statistical Computing, Vienna, Austria).

## 3 RESULTS

### 3.1 Characteristics of the cohort

From 2018 to 2021, there were 48,378 cases of cancer diagnosed in Manitoba (Table S1). The median age at diagnosis was 68 years, and 50.8% of the cohort were female. Over one half of individuals lived in Winnipeg (*n* = 27,726, 57.3%), 6791 (14.0%) lived in Prairie Mountain, 6225 (13.1%) lived in Southern, 5773 (11.9%) lived in Interlake‐Eastern, and 1753 (3.6%) lived in Northern.

### 3.2 Cancer incidence

**FIGURE 2:**
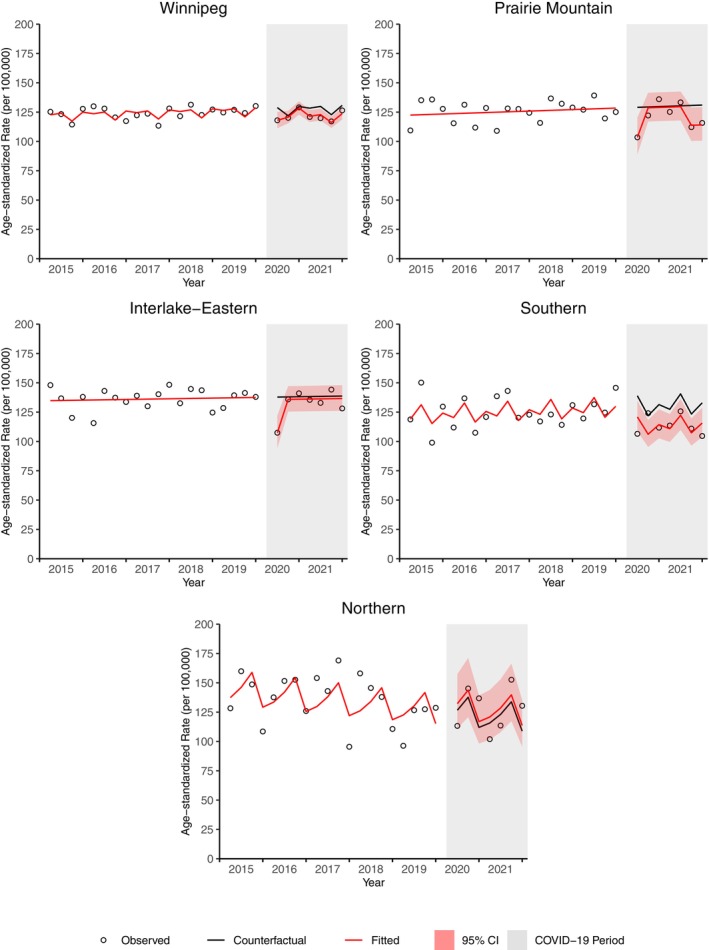
Age‐standardized incidence rate for all cancers by region from April 2020 to December 31, 2021.

**FIGURE 3:**
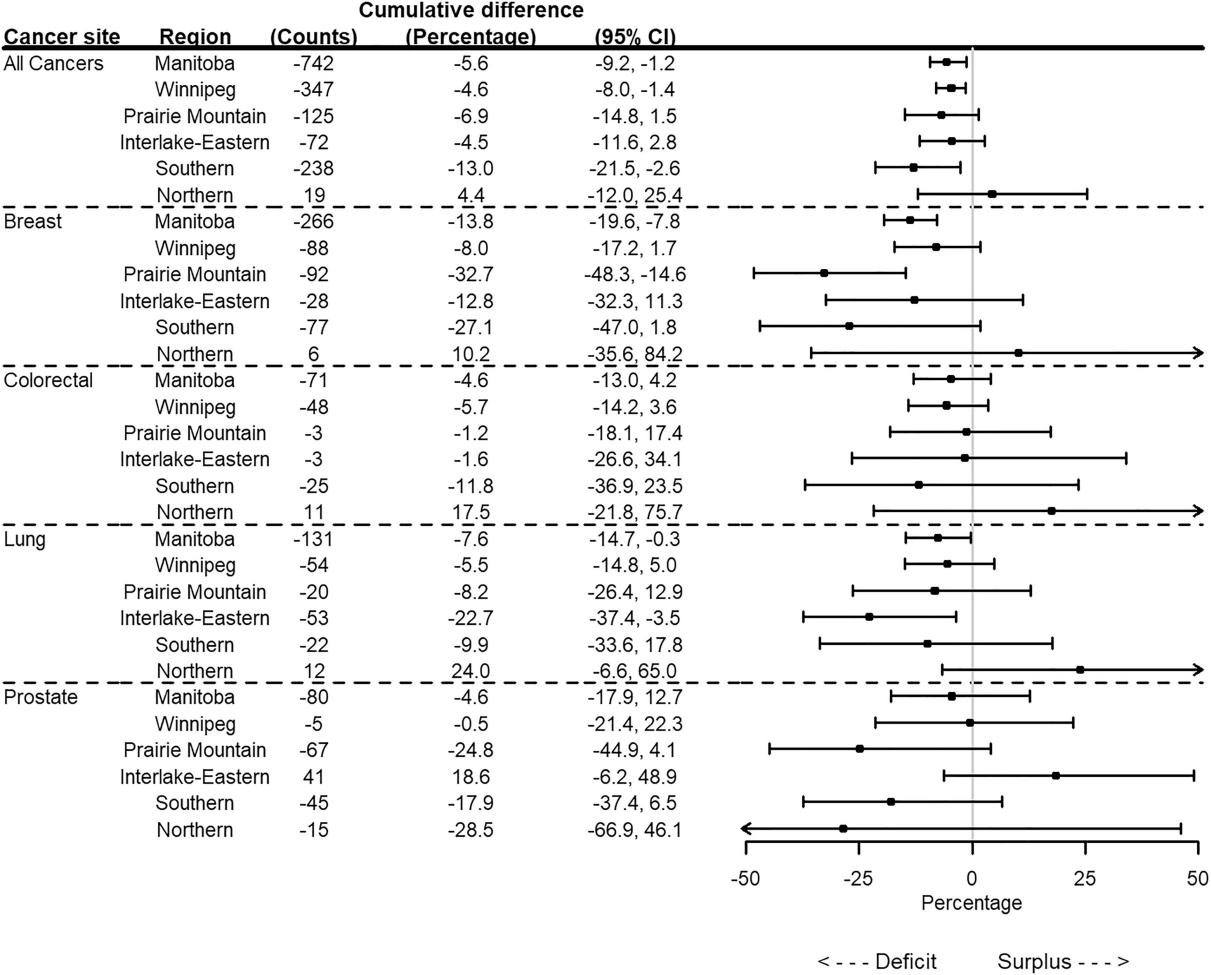
Estimated cumulative difference and percent cumulative difference between the fitted and predicted number of cancer cases by cancer site for Manitoba and by region as of December 31, 2021.

**FIGURE 4:**
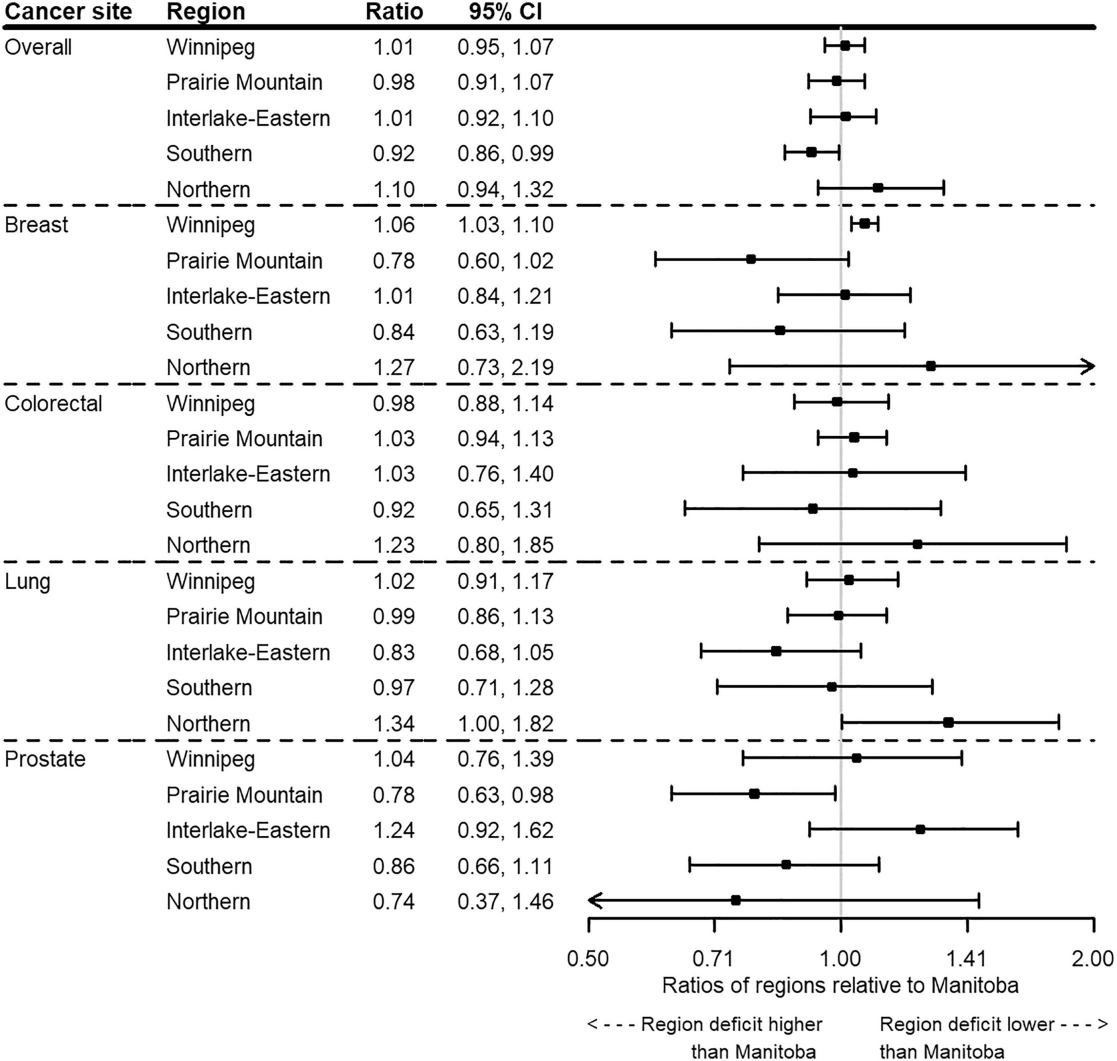
Ratio of cancer incidence by cancer site and region relative to Manitoba as of December 31, 2021.

All regions except the Northern region demonstrated a decrease in cancer incidence from April to June 2020 (Figure 2). Cancer incidence was significantly lower than expected in Prairie Mountain from July 2021 to December 2021, and in Southern from April 2020 to December 2021 (Table S2). As of December 2021, a significant deficit in cancer cases was observed for Winnipeg (4.6% deficit, 347 cases) and Southern (13.0% deficit, 238 cases) (Table S3; Figure 3). There was a non‐significant surplus of cases in the Northern region (4.4% surplus, 19 cases). Southern was the only region that had a significantly higher deficit in cases compared to Manitoba as a whole (ratio 0.92, 95% CI 0.86, 0.99) (Table S4; Figure 4).

### 3.3 Breast cancer incidence

**FIGURE 5:**
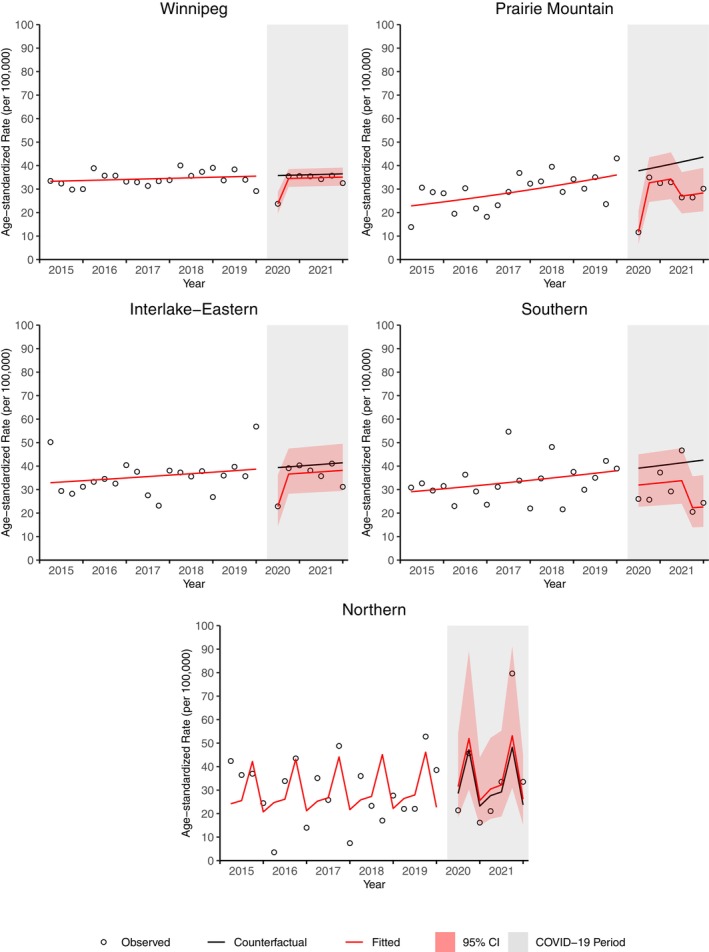
Age‐standardized breast cancer incidence rate for Manitoba and by region from April 2020 to December 2021.

Winnipeg, Prairie‐Mountain, and Interlake‐Eastern demonstrated a significant decrease in breast cancer incidence during the first quarter of the pandemic (April–June 2020) (Figure 5). Only Southern and Prairie Mountain demonstrated a significant decrease in breast cancer incidence during the delta wave in the fall of 2021. The percent deficit in breast cancer cases was 32.7% (92 cases) in Prairie Mountain and 27.1% (77 cases) in Southern. Compared to Manitoba, the ratio of the counterfactual incidence relative to the fitted incidence was 0.78 (95% CI 0.60, 1.02) and 0.84 (95% CI 0.63, 1.19) for Prairie Mountain and Southern, respectively.

### 3.4 Colorectal cancer incidence

**FIGURE 6:**
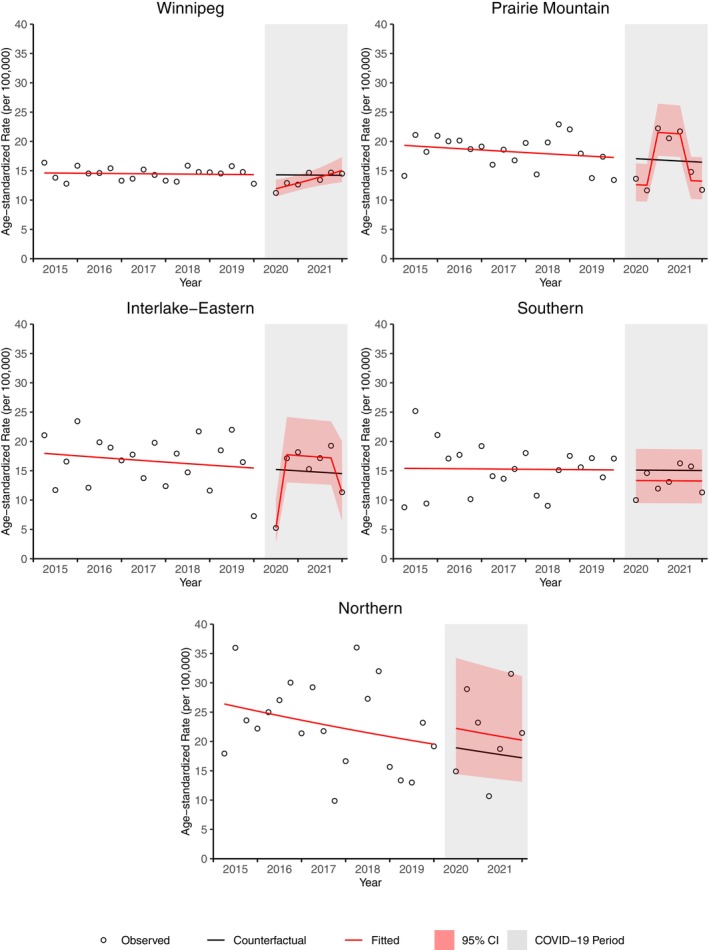
Age‐standardized colorectal cancer incidence rate for Manitoba and by region from April 2020 to December 2021.

Winnipeg demonstrated a significant decrease in colorectal cancer incidence from April to September 2020 while Interlake‐Eastern had a large decrease in the first pandemic quarter (Figure 6). In Prairie Mountain, colorectal cancer incidence was significantly higher than expected from October 2020 to June 2021. Southern had a sustained decrease in colorectal cancer incidence over the entire time period, although it was not significant. The cumulative percent deficit was highest in Southern (11.8% deficit, 25 cases), where the ratio of the counterfactual incidence relative to the observed fitted incidence was 0.92 (95% CI 0.65, 1.31) compared to Manitoba.

### 3.5 Lung cancer incidence

**FIGURE 7:**
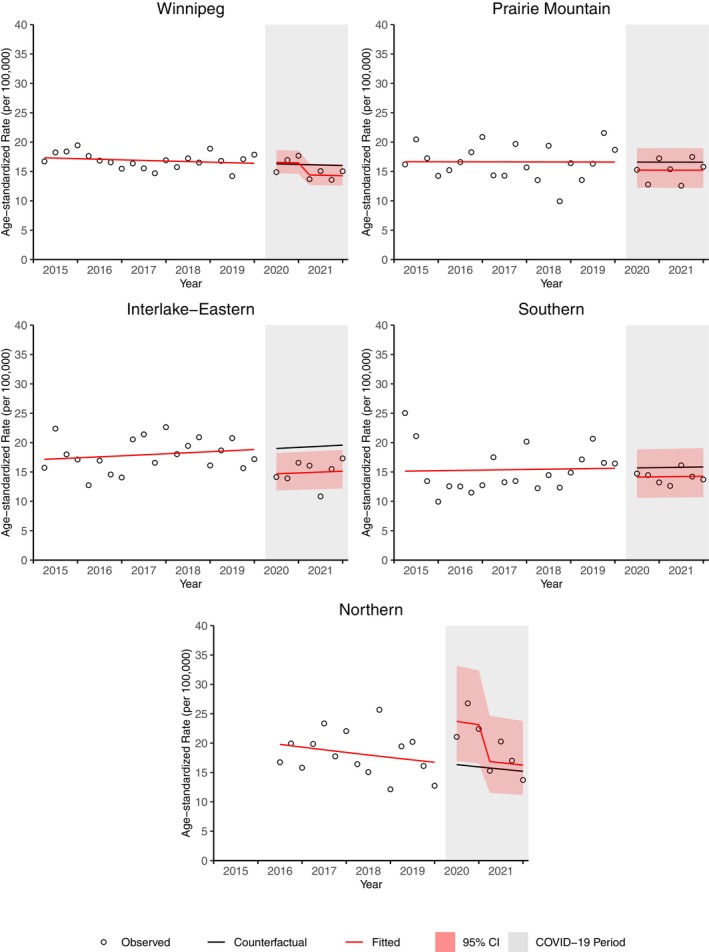
Age‐standardized lung cancer incidence rate for Manitoba and by region from April 2020 to December 2021.

Interlake‐Eastern demonstrated significantly lower lung cancer incidence relative to the counterfactual from April 2020 to December 2021 (Figure 7). Because of a few unusually high values during the pre‐COVID‐19 time period which greatly influenced the counterfactual, we excluded the first quarter of 2015 to the first quarter of 2016 from the analysis for the Northern region. Northern demonstrated a significant increase in lung cancer incidence from April to December 2020. The cumulative percent deficit was 22.7% (53 cases) in Interlake‐Eastern. Compared to Manitoba, the ratio of the counterfactual incidence relative to the fitted incidence was 0.83 (95% CI 0.68, 1.05) in Interlake‐Eastern and 1.34 (95% CI 1.00, 1.82) in Northern.

### 3.6 Prostate cancer incidence

**FIGURE 8:**
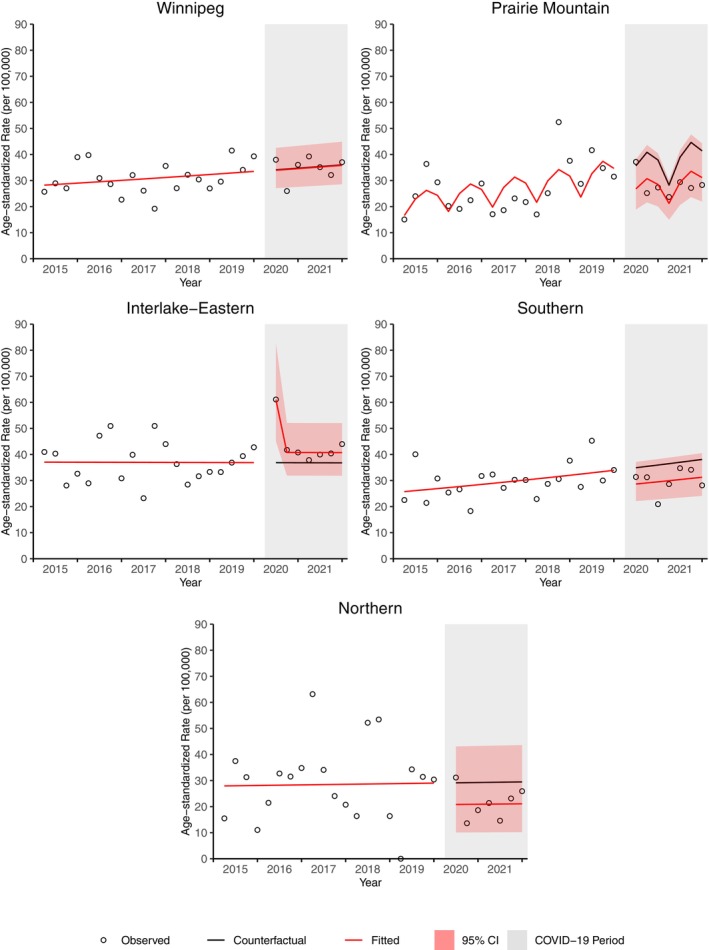
Age‐standardized prostate incidence rate for Manitoba and by region from April 2020 to December 2021.

Prairie Mountain, Southern, and Northern demonstrated a non‐significant decrease in prostate cancer incidence throughout the pandemic (Figure 8). Interlake‐Eastern demonstrated an increase in prostate cancer incidence over the entire time period. The largest cumulative percent deficit in prostate cancer cases occurred in Prairie Mountain (24.8% deficit, 67 cases) and Northern (28.5% deficit, 15 cases) regions. The cumulative percentage of cases increased in Interlake‐Eastern by 18.6% (41 cases). Compared to Manitoba, the ratio of the counterfactual incidence relative to the fitted incidence was 1.24 (95% CI 0.92, 1.62) in Interlake‐Eastern, 0.78 (95% CI 0.63, 0.98) in Prairie Mountain, and 0.86 (95% CI 0.65, 1.11) in Southern.

## 4 DISCUSSION

We found that the different regions of Manitoba demonstrated variability in cancer incidence during the first 21 months of the pandemic. Overall cancer incidence remained significantly lower than expected in Southern and Winnipeg. However, the 95% confidence limits for the Winnipeg ratio were substantially narrower than all other regions because more than 50% of Manitoba's population resides in Winnipeg, and the cumulative difference was relatively close to 0. Therefore, the cumulative difference for Winnipeg is not considered substantially lower than expected values. Outside of Winnipeg, Southern had the largest percent cumulative deficit in cancer cases, and was the only region that was significantly different than Manitoba as a whole. Southern experienced a high number of COVID‐19 cases and hospitalizations, particularly during the fall of 2021 during the Delta wave, which may have impacted the early detection of cancer.^11^

Unlike other regions of Manitoba, cancer incidence did not decrease in the Northern region and there was no cumulative deficit in the number of cancer cases. This finding may indicate that the COVID‐19‐related restrictions put in place to protect the residents of this region did not exacerbate existing disparities in health care access leading to a subsequent decline in the number of people newly diagnosed with cancer. However, it is important to note that the population of the Northern region and the number of cancer cases are small. Because of this, results must be interpreted with caution due to limited power.

We also found that cancer incidence by cancer type differed by region. The decrease in breast cancer incidence at the start of the pandemic seen in all regions except the Northern region is related to the suspension of the provincial breast screening program during the first few months of the pandemic.^28^ After the breast screening program returned to routine operations across the province,^28^ Prairie Mountain and Southern still had lower than expected breast cancer incidence. The decrease may be related to longer wait times for diagnostic bilateral mammograms following an abnormal screening mammogram. Breast cancers are frequently slow growing,^29^, ^30^, ^31^ so any delays in diagnosis may not lead to increases in cancer stage. Colorectal cancer incidence also decreased at the start of the pandemic in all regions except Northern. This is also likely related to reductions in the provincial colorectal cancer screening program as well as decreases in colonoscopy availability. Sustained decreases in colorectal cancer incidence were also seen in the Southern region, although they were not significant. The number of screening program fecal occult blood tests and/or colonoscopies may have been substantially lower than expected in the Southern region which, along with higher COVID‐19 incidence and death rates, may explain the lower CRC incidence.

Lung cancer incidence decreased significantly in Interlake‐Eastern during the Delta wave in the spring of 2021. We previously observed a decrease in lung cancer, but this occurred only among individuals 75 years of age and older.^6^ We speculated that part of the decrease may be due to the increased rate of COVID‐19 infections in Manitoba in the second and third waves leading to a higher number of deaths among individuals with undiagnosed lung cancer who were more vulnerable to severe COVID‐19 outcomes because of their age and comorbidities.^32^, ^33^ This is because individuals at the highest risk of COVID‐19‐related mortality (e.g., due to pre‐existing pulmonary or cardiac comorbidities) also have increased risk factors of developing lung cancer. Individuals may also have continued to be reluctant to seek healthcare or did not want to burden the health care system, especially for minor respiratory symptoms that were assumed to be COVID‐19 related.^34^, ^35^ Interlake‐Eastern does have the oldest population in the province; hence, the pandemic may have had a larger impact on lung cancer diagnoses in this region compared to others. However, because we adjusted for age in the analyses, the results may be related to other factors that interact with age such as comorbidity or functional health. By contrast, lung cancer incidence increased in the Northern region during 2020. Northern had the highest rate of COVID‐19 testing during the second and third waves, which may have resulted in incidental lung cancer diagnoses. Similar increases in lung cancer incidence have been found in other jurisdictions.^36^ The increase in lung cancer incidence should be interpreted with caution because of the small number of lung cancer cases and the wide CIs.

Prairie Mountain, Southern, and Northern demonstrated non‐significant decreases in prostate cancer incidence. Since prostate cancer is often diagnosed at an early stage^37^ and there is no organized, province‐wide screening program in place in Manitoba (the Canadian Task Force on Preventive Health Care recommends against screening males for prostate cancer with the PSA test^38^), this decrease could reflect a reduction in the rate of overdiagnosis for prostate cancer based on “routine” prostate‐specific antigen bloodwork conducted as a part of annual assessments by family medicine practitioners. Interlake‐Eastern had higher than expected prostate cancer incidence during the first quarter of the pandemic, but this trend was neither statistically significant nor sustained over time.

The strengths of this study include the use of a quasi‐experimental study design^13^ and an ITS analysis with a long pre‐intervention period, which permitted the evaluation of outcomes before the start of the COVID‐19 pandemic, as well as the inclusion of seasonality and interactions between COVID‐19 pandemic onset and time in the analysis. MCR data were complete to December 2021, so we were able to include the first three waves (21 months) of the COVID‐19 pandemic; the impact of subsequent waves has yet to be measured. However, our results must be interpreted within the Manitoba context as well as possible limitations due to small numbers and wide CIs, particularly in the Northern region. For example, we were not able to examine cervical cancer incidence by region because of small numbers, but this work could be done in areas with a larger population.

## 5 CONCLUSION

There was variability in cancer incidence across different regions of Manitoba during the first 21 months of the pandemic. Substantial deficits in breast and prostate cancers were found for the Prairie Mountain and Southern regions. Because many breast and prostate cancers are frequently slow growing and prostate cancers are often treated with active surveillance, these deficits might not lead to increases in cancer stage. Cancer incidence did not significantly decrease in Northern Manitoba, an area that experiences challenges regarding disparities in health care and health status. Measuring and understanding these differences at a regional level are important when directing pandemic recovery efforts and planning for future health care disruptions.

## AUTHOR CONTRIBUTIONS

**Kathleen M. Decker:** Conceptualization (lead); formal analysis (equal); funding acquisition (lead); methodology (equal); project administration (lead); resources (lead); supervision (lead); validation (equal); visualization (equal); writing – original draft (lead); writing – review and editing (lead). **Allison Feely:** Conceptualization (supporting); data curation (supporting); formal analysis (equal); methodology (equal); validation (equal); visualization (equal); writing – original draft (equal); writing – review and editing (equal). **Oliver Bucher:** Conceptualization (equal); data curation (equal); formal analysis (equal); funding acquisition (equal); methodology (equal); validation (equal); visualization (equal); writing – original draft (equal); writing – review and editing (equal). **Piotr Czaykowski:** Funding acquisition (equal); writing – review and editing (equal). **Pamela Hebbard:** Funding acquisition (equal); writing – review and editing (equal). **Julian O. Kim:** Funding acquisition (equal); writing – review and editing (equal). **Harminder Singh:** Funding acquisition (equal); writing – review and editing (equal). **Maclean Thiessen:** Funding acquisition (equal); writing – review and editing (equal). **Marshall Pitz:** Funding acquisition (equal); writing – review and editing (equal). **Grace Musto:** Data curation (equal); formal analysis (equal); methodology (equal); validation (equal); visualization (equal); writing – review and editing (equal). **Katie Galloway:** Data curation (equal); formal analysis (equal); methodology (equal); validation (equal); visualization (equal); writing – review and editing (equal). **Pascal Lambert:** Conceptualization (equal); data curation (equal); formal analysis (lead); funding acquisition (equal); methodology (equal); validation (equal); visualization (equal); writing – original draft (equal); writing – review and editing (equal).

## CONFLICT OF INTEREST STATEMENT

The authors declare no conflict of interest. The funders had no role in the design of the study, in the collection, analyses, or interpretation of data, in the writing of the manuscript, or in the decision to publish the results.

## ETHICS STATEMENT

The study was approved by the University of Manitoba's Health Research Ethics Board (HS23979; H2020:264) and CancerCare Manitoba's (CCMB) Research and Resource Impact Committee (2020‐14).

## Supporting information


Table S1.

## Data Availability

The data that support the findings of this study are not publicly available to ensure and maintain the privacy and confidentiality of individuals' health information. Requests for data may be made to the appropriate data stewards (CancerCare Manitoba's Research and Resource Impact Committee).
