# Supplementary material for: Cancer incidence during the COVID‐19 pandemic by region of residence in Manitoba, Canada: A cancer registry‐based interrupted time series study
Source: Cancer Med. 2023 Nov 16;12(23):21465–79. doi: 10.1002/cam4.6698 (PMC10726851; doi:10.1002/cam4.6698)
Supplement: Supplementary file 1 — Table S1. [file CAM4-12-21465-s001.docx]

**Supplementary Material**

**Table 1. Characteristic of individuals diagnosed cancer by cancer type in Manitoba, January 2015 to December 2021**

| **Cancer Site** | **Region** | **Cases** | **Age at diagnosis** | **Sex** | |
| --- | --- | --- | --- | --- | --- |
|  |  |  | **Median** | **Male** | **Female** |
| **All Cancers** | Winnipeg | 27,726 | 68 | 13,652 (49.2) | 14,073 (50.8) |
|  | Prairie Mountain | 6,791 | 70 | 3,487 (51.4) | 3,304 (48.6) |
|  | Interlake-Eastern | 5,773 | 68 | 3,099 (53.7) | 2,674 (46.3) |
|  | Southern | 6,335 | 68 | 3,253 (51.4) | 3,081 (48.6) |
|  | Northern | 1,753 | 64 | 913 (52.1) | 840 (47.9) |
| **Breast** | Winnipeg | 3,979 | 65 | 0 (0.0) | 3,979 (100.0) |
|  | Prairie Mountain | 782 | 68 | 0 (0.0) | 782 (100.0) |
|  | Interlake-Eastern | 738 | 65 | 0 (0.0) | 738 (100.0) |
|  | Southern | 837 | 64 | 0 (0.0) | 837 (100.0) |
|  | Northern | 212 | 62 | 0 (0.0) | 212 (100.0) |
| **Colorectal** | Winnipeg | 3,177 | 70 | 1,742 (54.8) | 1,435 (45.2) |
|  | Prairie Mountain | 980 | 71 | 533 (54.4) | 447 (45.6) |
|  | Interlake-Eastern | 688 | 69 | 385 (56.0) | 303 (44.0) |
|  | Southern | 756 | 69 | 425 (56.2) | 331 (43.8) |
|  | Northern | 287 | 64 | 173 (60.3) | 114 (39.7) |
| **Lung** | Winnipeg | 3,772 | 72 | 1,760 (46.7) | 2,012 (53.3) |
|  | Prairie Mountain | 932 | 72 | 432 (46.4) | 500 (53.6) |
|  | Interlake-Eastern | 753 | 71 | 381 (50.6) | 372 (49.4) |
|  | Southern | 787 | 72 | 421 (53.5) | 366 (46.5) |
|  | Northern | 233 | 68 | 108 (46.4) | 125 (53.6) |
| **Prostate** | Winnipeg | 3,315 | 69 | 3,315 (100.0) | 0 (0.0) |
|  | Prairie Mountain | 749 | 72 | 749 (100.0) | 0 (0.0) |
|  | Interlake-Eastern | 864 | 69 | 864 (100.0) | 0 (0.0) |
|  | Southern | 753 | 70 | 753 (100.0) | 0 (0.0) |
|  | Northern | 168 | 68 | 168 (100.0) | 0 (0.0) |

**Table 2. Ratios and 95% confidence intervals (CI) between fitted and predicted cancer incidence, April 2020 to December 2021**

| **Region** | **Year and Quarter** | **All sites** | **Breast** | **Lung** | **Colorectal** | **Prostate** |
| --- | --- | --- | --- | --- | --- | --- |
| **Manitoba** | **2020** |  |  |  |  |  |
|  | Apr-Jun | 0.88 (0.82, 0.94) | 0.61 (0.54, 0.69) | 0.97 (0.89, 1.06) | 0.72 (0.61, 0.85) | 0.95 (0.81, 1.12) |
|  | Jul-Sep | 0.98 (0.93, 1.03) | 0.92 (0.86, 0.99) | 0.97 (0.89, 1.06) | 0.99 (0.90, 1.09) | 0.95 (0.81, 1.12) |
|  | Oct-Dec | 0.98 (0.93, 1.03) | 0.92 (0.86, 0.99) | 0.97 (0.89, 1.06) | 0.99 (0.90, 1.09) | 0.95 (0.81, 1.12) |
|  | **2021** |  |  |  |  |  |
|  | Jan-Mar | 0.94 (0.90, 0.99) | 0.92 (0.86, 0.99) | 0.89 (0.81, 0.98) | 0.99 (0.90, 1.09) | 0.95 (0.81, 1.12) |
|  | Apr-Jun | 0.94 (0.90, 0.99) | 0.92 (0.86, 0.99) | 0.89 (0.81, 0.98) | 0.99 (0.90, 1.09) | 0.95 (0.81, 1.12) |
|  | Jul-Sep | 0.94 (0.90, 0.99) | 0.92 (0.86, 0.99) | 0.89 (0.81, 0.98) | 0.99 (0.90, 1.09) | 0.95 (0.81, 1.12) |
|  | Oct-Dec | 0.94 (0.90, 0.99) | 0.81 (0.71, 0.91) | 0.89 (0.81, 0.98) | 0.99 (0.90, 1.09) | 0.95 (0.81, 1.12) |
| **Winnipeg** | **2020** |  |  |  |  |  |
|  | Apr-Jun | 0.92 (0.86, 0.97) | 0.66 (0.55, 0.80) | 1.02 (0.90, 1.15) | 0.83 (0.74, 0.94) | 1.00 (0.79, 1.25) |
|  | Jul-Sep | 0.99 (0.95, 1.03) | 0.96 (0.86, 1.07) | 1.02 (0.90, 1.15) | 0.87 (0.78, 0.97) | 1.00 (0.79, 1.25) |
|  | Oct-Dec | 0.99 (0.95, 1.03) | 0.96 (0.86, 1.07) | 1.02 (0.90, 1.15) | 0.90 (0.82, 1.00) | 1.00 (0.79, 1.25) |
|  | **2021** |  |  |  |  |  |
|  | Jan-Mar | 0.95 (0.91, 0.99) | 0.96 (0.86, 1.07) | 0.89 (0.79, 1.01) | 0.94 (0.85, 1.04) | 1.00 (0.79, 1.25) |
|  | Apr-Jun | 0.95 (0.91, 0.99) | 0.96 (0.86, 1.07) | 0.89 (0.79, 1.01) | 0.98 (0.88, 1.09) | 1.00 (0.79, 1.25) |
|  | Jul-Sep | 0.95 (0.91, 0.99) | 0.96 (0.86, 1.07) | 0.89 (0.79, 1.01) | 1.02 (0.90, 1.15) | 1.00 (0.79, 1.25) |
|  | Oct-Dec | 0.95 (0.91, 0.99) | 0.96 (0.86, 1.07) | 0.89 (0.79, 1.01) | 1.06 (0.92, 1.22) | 1.00 (0.79, 1.25) |
| **Prairie Mountain** | **2020** |  |  |  |  |  |
|  | Apr-Jun | 0.80 (0.69, 0.93) | 0.31 (0.17, 0.55) | 0.92 (0.74, 1.14) | 0.74 (0.57, 0.95) | 0.75 (0.53, 1.07) |
|  | Jul-Sep | 0.99 (0.90, 1.09) | 0.84 (0.63, 1.12) | 0.92 (0.74, 1.14) | 0.74 (0.57, 0.95) | 0.75 (0.53, 1.07) |
|  | Oct-Dec | 0.99 (0.90, 1.09) | 0.84 (0.63, 1.12) | 0.92 (0.74, 1.14) | 1.28 (1.04, 1.57) | 0.75 (0.53, 1.07) |
|  | **2021** |  |  |  |  |  |
|  | Jan-Mar | 0.99 (0.90, 1.09) | 0.84 (0.63, 1.12) | 0.92 (0.74, 1.14) | 1.28 (1.04, 1.57) | 0.75 (0.53, 1.07) |
|  | Apr-Jun | 0.99 (0.90, 1.09) | 0.65 (0.47, 0.89) | 0.92 (0.74, 1.14) | 1.28 (1.04, 1.57) | 0.75 (0.53, 1.07) |
|  | Jul-Sep | 0.87 (0.77, 0.99) | 0.65 (0.47, 0.89) | 0.92 (0.74, 1.14) | 0.80 (0.61, 1.05) | 0.75 (0.53, 1.07) |
|  | Oct-Dec | 0.87 (0.77, 0.99) | 0.65 (0.47, 0.89) | 0.92 (0.74, 1.14) | 0.80 (0.61, 1.05) | 0.75 (0.53, 1.07) |
| **Interlake Eastern** | **2020** |  |  |  |  |  |
|  | Apr-Jun | 0.78 (0.68, 0.89) | 0.58 (0.37, 0.92) | 0.77 (0.62, 0.96) | 0.35 (0.18, 0.67) | 1.66 (1.22, 2.24) |
|  | Jul-Sep | 0.98 (0.91, 1.07) | 0.92 (0.71, 1.20) | 0.77 (0.62, 0.96) | 1.17 (0.86, 1.60) | 1.11 (0.87, 1.41) |
|  | Oct-Dec | 0.98 (0.91, 1.07) | 0.92 (0.71, 1.20) | 0.77 (0.62, 0.96) | 1.17 (0.86, 1.60) | 1.11 (0.87, 1.41) |
|  | **2021** |  |  |  |  |  |
|  | Jan-Mar | 0.98 (0.91, 1.07) | 0.92 (0.71, 1.20) | 0.77 (0.62, 0.96) | 1.17 (0.86, 1.60) | 1.11 (0.87, 1.41) |
|  | Apr-Jun | 0.98 (0.91, 1.07) | 0.92 (0.71, 1.20) | 0.77 (0.62, 0.96) | 1.17 (0.86, 1.60) | 1.11 (0.87, 1.41) |
|  | Jul-Sep | 0.98 (0.91, 1.07) | 0.92 (0.71, 1.20) | 0.77 (0.62, 0.96) | 1.17 (0.86, 1.60) | 1.11 (0.87, 1.41) |
|  | Oct-Dec | 0.98 (0.91, 1.07) | 0.92 (0.71, 1.20) | 0.77 (0.62, 0.96) | 0.78 (0.44, 1.38) | 1.11 (0.87, 1.41) |
| **Southern** | **2020** |  |  |  |  |  |
|  | Apr-Jun | 0.87 (0.78, 0.97) | 0.82 (0.58, 1.15) | 0.90 (0.68, 1.20) | 0.88 (0.63, 1.24) | 0.82 (0.63, 1.06) |
|  | Jul-Sep | 0.87 (0.78, 0.97) | 0.82 (0.58, 1.15) | 0.90 (0.68, 1.20) | 0.88 (0.63, 1.24) | 0.82 (0.63, 1.06) |
|  | Oct-Dec | 0.87 (0.78, 0.97) | 0.82 (0.58, 1.15) | 0.90 (0.68, 1.20) | 0.88 (0.63, 1.24) | 0.82 (0.63, 1.06) |
|  | **2021** |  |  |  |  |  |
|  | Jan-Mar | 0.87 (0.78, 0.97) | 0.82 (0.58, 1.15) | 0.90 (0.68, 1.20) | 0.88 (0.63, 1.24) | 0.82 (0.63, 1.06) |
|  | Apr-Jun | 0.87 (0.78, 0.97) | 0.82 (0.58, 1.15) | 0.90 (0.68, 1.20) | 0.88 (0.63, 1.24) | 0.82 (0.63, 1.06) |
|  | Jul-Sep | 0.87 (0.78, 0.97) | 0.53 (0.33, 0.85) | 0.90 (0.68, 1.20) | 0.88 (0.63, 1.24) | 0.82 (0.63, 1.06) |
|  | Oct-Dec | 0.87 (0.78, 0.97) | 0.53 (0.33, 0.85) | 0.90 (0.68, 1.20) | 0.88 (0.63, 1.24) | 0.82 (0.63, 1.06) |
| **Northern** | **2020** |  |  |  |  |  |
|  | Apr-Jun | 1.04 (0.88, 1.24) | 1.10 (0.64, 1.89) | 1.45 (1.05, 2.03) | 1.17 (0.76, 1.81) | 0.71 (0.35, 1.48) |
|  | Jul-Sep | 1.04 (0.88, 1.24) | 1.10 (0.64, 1.89) | 1.45 (1.05, 2.03) | 1.17 (0.76, 1.81) | 0.71 (0.35, 1.48) |
|  | Oct-Dec | 1.04 (0.88, 1.24) | 1.10 (0.64, 1.89) | 1.45 (1.05, 2.03) | 1.17 (0.76, 1.81) | 0.71 (0.35, 1.48) |
|  | **2021** |  |  |  |  |  |
|  | Jan-Mar | 1.04 (0.88, 1.24) | 1.10 (0.64, 1.89) | 1.07 (0.73, 1.56) | 1.17 (0.76, 1.81) | 0.71 (0.35, 1.48) |
|  | Apr-Jun | 1.04 (0.88, 1.24) | 1.10 (0.64, 1.89) | 1.07 (0.73, 1.56) | 1.17 (0.76, 1.81) | 0.71 (0.35, 1.48) |
|  | Jul-Sep | 1.04 (0.88, 1.24) | 1.10 (0.64, 1.89) | 1.07 (0.73, 1.56) | 1.17 (0.76, 1.81) | 0.71 (0.35, 1.48) |
|  | Oct-Dec | 1.04 (0.88, 1.24) | 1.10 (0.64, 1.89) | 1.07 (0.73, 1.56) | 1.17 (0.76, 1.81) | 0.71 (0.35, 1.48) |

Abbreviations: CI, confidence interval

**Table 3. Estimated cumulative fitted and counterfactual number of cancer cases, difference, and percent difference by site and region** **as of December 31, 2021**

| **Region** | **Cancer site** | **Cumulative fitted cancer cases (n)** | **Cumulative counterfactual cancer cases (n)** | **Cumulative difference** | **Percent cumulative difference* (95% CI)** |
| --- | --- | --- | --- | --- | --- |
| **Manitoba** | All | 12,457 | 13,200 | -742 | -5.6 (-9.2, -1.2) |
|  | Breast | 1,660 | 1,925 | -266 | -13.8 (-19.6, -7.8) |
|  | Lung | 1,600 | 1,730 | -131 | -7.6 (-14.7, -0.3) |
|  | Colorectal | 1,466 | 1,537 | -71 | -4.6 (-13.0, 4.2) |
|  | Prostate | 1,674 | 1,754 | -80 | -4.6 (-17.9, 12.7) |
|  |  |  |  |  |  |
| **Winnipeg** | All | 7,192 | 7,539 | -347 | -4.6 (-8.0, -1.4) |
|  | Breast | 1,008 | 1,096 | -88 | -8.0 (-17.2, 1.7) |
|  | Lung | 930 | 984 | -54 | -5.5 (-14.8, 5.0) |
|  | Colorectal | 797 | 845 | -48 | -5.7 (-14.2, 3.6) |
|  | Prostate | 965 | 970 | -5 | -0.5 (-21.4, 22.3) |
|  |  |  |  |  |  |
| **Prairie Mountain** | All | 1,705 | 1,830 | -125 | -6.9 (-14.8, 1.5) |
|  | Breast | 189 | 281 | -92 | -32.7 (-48.3, -14.6) |
|  | Lung | 226 | 246 | -20 | -8.2 (-26.4, 12.9) |
|  | Colorectal | 239 | 242 | -3 | -1.2 (-18.1, 17.4) |
|  | Prostate | 204 | 271 | -67 | -24.8 (-44.9, 4.1) |
|  |  |  |  |  |  |
| **Interlake Eastern** | All | 1,517 | 1,589 | -72 | -4.5 (-11.6, 2.8) |
|  | Breast | 193 | 221 | -28 | -12.8 (-32.3, 11.3) |
|  | Lung | 180 | 233 | -53 | -22.7 (-37.4, -3.5) |
|  | Colorectal | 171 | 174 | -3 | -1.6 (-26.6, 34.1) |
|  | Prostate | 264 | 222 | 41 | 18.6 (-6.2, 48.9) |
|  |  |  |  |  |  |
| **Southern** | All | 1,591 | 1,829 | -238 | -13.0 (-21.5, -2.6) |
|  | Breast | 207 | 283 | -77 | -27.1 (-47.0, 1.8) |
|  | Lung | 202 | 224 | -22 | -9.9 (-33.6, 17.8) |
|  | Colorectal | 184 | 209 | -25 | -11.8 (-36.9, 23.5) |
|  | Prostate | 208 | 253 | -45 | -17.9 (-37.4, 6.5) |
|  |  |  |  |  |  |
| **Northern** | All | 451 | 432 | 19 | 4.4 (-12.0, 25.4) |
|  | Breast | 63 | 57 | 6 | 10.2 (-35.6, 84.2) |
|  | Lung | 63 | 51 | 12 | 24.0 (-6.6, 65.0) |
|  | Colorectal | 74 | 63 | 11 | 17.5 (-21.8, 75.7) |
|  | Prostate | 38 | 54 | -15 | -28.5 (-66.9, 46.1) |

* A minus sign indicates a deficit and a plus sign indicates a surplus.

Abbreviations: CI, confidence interval

**Table 4. Ratio of the percent cumulative difference in the number of cancer cases by region and cancer site compared to Manitoba as of December 31, 2021**

| **Cancer site** | **Region** | **Ratio (95% CI)*** |
| --- | --- | --- |
| **All sites** | Winnipeg | 1.01 (0.95, 1.07) |
|  | Prairie Mountain | 0.99 (0.91, 1.07) |
|  | Interlake Eastern | 1.01 (0.92, 1.10) |
|  | Southern | 0.92 (0.86, 0.99) |
|  | Northern | 1.11 (0.94, 1.32) |
|  |  |  |
| **Breast** | Winnipeg | 1.07 (1.03, 1.10) |
|  | Prairie Mountain | 0.78 (0.60, 1.02) |
|  | Interlake Eastern | 1.01 (0.84, 1.21) |
|  | Southern | 0.85 (0.63, 1.19) |
|  | Northern | 1.28 (0.74, 2.19) |
|  |  |  |
| **Lung** | Winnipeg | 1.02 (0.91, 1.17) |
|  | Prairie Mountain | 0.99 (0.86, 1.13) |
|  | Interlake Eastern | 0.84 (0.68, 1.05) |
|  | Southern | 0.97 (0.71, 1.28) |
|  | Northern | 1.34 (1.00, 1.82) |
|  |  |  |
| **Colorectal** | Winnipeg | 0.99 (0.88, 1.14) |
|  | Prairie Mountain | 1.04 (0.94, 1.13) |
|  | Interlake Eastern | 1.03 (0.76, 1.40) |
|  | Southern | 0.92 (0.65, 1.31) |
|  | Northern | 1.23 (0.80, 1.85) |
|  |  |  |
| **Prostate** | Winnipeg | 1.04 (0.76, 1.39) |
|  | Prairie Mountain | 0.79 (0.63, 0.98) |
|  | Interlake Eastern | 1.24 (0.92, 1.62) |
|  | Southern | 0.86 (0.66, 1.11) |
|  | Northern | 0.75 (0.37, 1.46) |

*Manitoba is the denominator

Abbreviations: CI, confidence interval
