# Supplementary material for: Cancer incidence during the COVID‐19 pandemic by region of residence in Manitoba, Canada: A cancer registry‐based interrupted time series study
Source: Cancer Med. 2023 Nov 16;12(23):21465–79. doi: 10.1002/cam4.6698 (PMC10726851; doi:10.1002/cam4.6698)
Supplement: Supplementary file 2 [file CAM4-12-21465-s002.docx]

**eAppendix A. Cancer types included in each category and International Classification of Diseases for Oncology version 3 (ICD-O-3) codes**

| **Category** | **Cancer sites** | **ICD-O-3 codes** |
| --- | --- | --- |
| **Breast** | Female breast | C500-C509 |
| **Lung** | Small cell and non-small cell | C340-C349 |
| **Prostate** | Prostate | C619 |
| **Colon** | Colon | C180-189, C260 |
| **Rectal** | Rectal and rectosigmoid | C199, C209 |
| **Hematologic** | Hodgkin lymphoma | 9650-9667 |
|  | Non-Hodgkin lymphoma | 9590-9597, 9670-9671, 9673, 9675, 9678-9680, 9684, 9687-9691, 9695, 9698-9702, 9705, 9708-9709, 9712, 9714-9719, 9724-9729, 9735, 9737, 9738,  9811-9818 (except C420, C421, C424)  9823 (except C420, C421, C424)  9827 (except C420, C421, C424)  9837 (except C420, C421, C424) |
|  | Myeloma | 9731-9732, 9734 |
|  | Acute lymphocytic leukemia | 9826, 9835-9836  9811-9818 (only C421)  9837 (only C421) |
|  | Chronic lymphocytic leukemia | 9823 (only C421, if SLL C77) |
|  | Acute myeloid leukemia | 9840, 9861, 9865-9867, 9869, 9871-9874, 9895-9897, 9898, 9910-9911, 9920 |
|  | Acute monocytic leukemia | 9891 |
|  | Chronic myeloid leukemia | 9863, 9875-9876, 9945-9946 |
|  | Other leukemia | 9733, 9742, 9800, 9801, 9805-9809, 9820, 9831-9834, 9860, 9870, 9930, 9931, 9940, 9948, 9963, 9964  9827 (only C421) |
| **Urinary** | Bladder | C670-C679 |
|  | Kidney and renal pelvis | C649, C659 |
|  | Ureter | C669 |
|  | Other urinary system | C680-689 |
| **Unknown primary** | Unknown primary | 9740-9741, 9750-9769, 9950, 9960-9962, 9965-9967, 9970-9971, 9975, 9980, 9982-9987, 9989,9991-9992,  C420-C424 (except 9050-9055, 9140, 9590-9992)  C760-C768 (except 9050-9055, 9140, 9590-9992)  C770-C779 (except 9050-9055, 9140, 9590-9992)  C809 (except 9050-9055, 9140, 9590-9992) |
| **Head and neck** | Buccal cavity and pharynx | C00-C14 |
|  | Larynx | C320-C329 |
|  | Other non-lung respiratory | C300-C301, C310-C319, C384, C339, C381-C383, C388, C390-C399 |
| **Brain and central nervous system** | Brain | C710-C719 |
|  | Other nervous system | C710-C719 (953), C700-C709, C720-C729 |
| **Gynecologic** | Cervix uteri | C530-C539 |
|  | Corpus uteri | C540-C549 |
|  | Uterus, NOS | C559 |
|  | Ovary | C569 |
|  | Other female genital system | C529, C510-C519, C570-C589 |
| **Category** | **Cancer sites** | **ICD-O-3 codes** |
| **Other digestive** | Esophagus | C150-C159 |
|  | Stomach | C160-C169 |
|  | Small intestine | C170-C179 |
|  | Anus | C210-C212, C218 |
|  | Liver | C220 |
|  | Gallbladder | C239 |
|  | Other digestive system | C240-C249, C221, C480, C481-C482, C268-C269, C488 |
| **Melanoma** | Melanoma | C440-C449 (8720-8790) |
| **Pancreatic** | Pancreatic | C250-C259 |
| **Endocrine** | Thyroid | C739 |
|  | Other endocrine | C379, C740-C749, C750-C759 |
| **Other** | Bones and joints | C400-C419 |
|  | Soft tissue (including heart) | C380, C470-C479, C490-C499 |
|  | Mesothelioma | 9050-9055 |
|  | Kaposi sarcoma | 9140 |
|  | Eye | C690-C699 |
|  | Male breast | C500-C509 |
|  | Testis | C620-C629 |
|  | Penis | C600-C609 |
|  | Other male genital system | C630-C639 |
